# Coffee extract inhibits adipogenesis in 3T3-L1 preadipocyes by interrupting insulin signaling through the downregulation of IRS1

**DOI:** 10.1371/journal.pone.0173264

**Published:** 2017-03-10

**Authors:** Chihiro Maki, Megumi Funakoshi-Tago, Ryohei Aoyagi, Fumihito Ueda, Masaki Kimura, Kenji Kobata, Kenji Tago, Hiroomi Tamura

**Affiliations:** 1 Graduate School of Pharmaceutical Sciences, Keio University, Minato-ku, Tokyo, Japan; 2 Faculty of Pharmaceutical Sciences, Josai University, Sakado, Saitama, Japan; 3 Division of Structural Biochemistry, Department of Biochemistry, Jichi Medical University, Shimotsuke-shi, Tochigi-ken, Japan; USDA-ARS, UNITED STATES

## Abstract

Although epidemiological data have indicated that a strong negative association exists between coffee consumption and the prevalence of obesity-associated diseases, the molecular mechanisms by which coffee intake prevents obesity-associated diseases has not yet been elucidated. In this study, we found that coffee intake significantly suppressed high-fat diet (HFD)-induced metabolic alternations such as increases in body weight and the accumulation of adipose tissue, and up-regulation of glucose, free fatty acid, total cholesterol and insulin levels in the blood. We also found that coffee extract significantly inhibited adipogenesis in 3T3-L1 preadipocytes. In the early phase of adipogenesis, 3T3-L1 cells treated with coffee extract displayed the retardation of cell cycle entry into the G2/M phase called as mitotic clonal expansion (MCE). Coffee extract also inhibited the activation of CCAAT/enhancer-binding protein β (C/EBPβ) by preventing its phosphorylation by ERK. Furthermore, the coffee extract suppressed the adipogenesis-related events such as MCE and C/EBPβ activation through the down-regulation of insulin receptor substrate 1 (IRS1). The stability of the IRS1 protein was markedly decreased by the treatment with coffee extract due to proteasomal degradation. These results have revealed an anti-adipogenic function for coffee intake and identified IRS1 as a novel target for coffee extract in adipogenesis.

## Introduction

Obesity is a major public health issue worldwide and is a significant risk factor for many serious metabolic diseases such as heart disease, type 2 diabetes, atherosclerosis, and cancer. Obesity arises from an imbalance in energy intake and energy expenditure. It is characterized by an increased amount of white adipose tissue (WAT), which is caused by an elevated adipocyte cell number or the disordered accumulation of lipid droplets in adipocytes. The accumulation of excessive amounts of lipids in adipocytes increases the triglyceride content in plasma and tissues such as the liver and muscle, which leads to pathological dysfunctions [1–3]. Therefore, in order to prevent obesity and develop effective strategies to control lipid metabolism, it is important to understand the molecular mechanisms that is responsible for adipogenesis, and search the bioactive substances preventing adipogenensis for controlling the symptoms of metabolic diseases. Interestingly, numerous epidemiological studies have reported that high coffee consumption decreased the risk of several chronic diseases including type 2 diabetes, cardiovascular disease, and cancer [4–6]. Moreover, previous studies have demonstrated that coffee extract inhibited fat accumulation, body weight, obesity and insulin resistance in obese mice [7–9]. These findings indicate that coffee consumption is beneficial for the prevention of obesity and obesity-associated metabolic diseases. However, since all previous studies have been observational and experimental evidence is limited, the molecular mechanisms by which coffee intake reduces the risk of various diseases currently remain unclear.

The murine fibroblast cell line, 3T3-L1 has been utilized as an *in vitro* model of adipogenesis [10, 11]. When treated with a combination of differentiation inducers called MDI, which consists of 3-isobutyl-1-methylxanthine (IBMX), dexamethasone, and insulin, 3T3-L1 cells differentiate into adipocytes, and this is regulated by the sequential expression of transcription factors such as the C/EBPs and peroxisome proliferator-activated receptor γ (PPARγ). During 3T3-L1 adipocyte differentiation, C/EBPδ and C/EBPβ are induced early by dexamethasone and IBMX, respectively [12–15]. C/EBPβ is responsible for mitotic clonal expansion (MCE), the process of MDI-induced mitogenic responses that induce approximately two rounds of cell division, which is critical for adipogenesis [16]. In terminal adipocyte differentiation, C/EBPδ and C/EBPβ cooperatively induce the expression of PPARγ and C/EBPα, master transcription factors for terminal adipocyte differentiation [12–15]. PPARγ and C/EBPα coordinately induce the expression of adipogenesis-related genes, including *Fabp4* and *Glut4*, which are functional markers for adipocytes [17–19].

Previously, we found that coffee extract inhibited adipocyte differentiation in 3T3-L1 cells via preventing the expression of *Pparγ* mRNA; however, inhibitory mechanism on *Pparγ* expression by coffee extract remained unclear [20]. In the present study, we investigated the molecular mechanisms by which coffee extract counteracts adipogenesis in 3T3-L1 preadipocytes to understand the anti-obesity effect of coffee extract.

## Materials and methods

### Preparation of coffee extract

Roasted coffee powder (Columbia Arabica) was obtained from Starbucks Coffee Japan (Tokyo, Japan). Coffee extract was prepared by a common method (drip style), in which 8 g of powder was poured with 140 mL hot water (95°C). The extract was then filtered through a paper filter (Mellita, Minden, Germany), divided into small aliquots, and stored at −80°C until used. Undiluted extract, with a dry weight of 8.4 mg/mL, was assigned a concentration of 100% (v/v) as previously reported [20].

### Animals and composition of the diet and coffee extract

Male C57BL/6JJmsSlc mice (4 weeks old) were obtained from Sankyo Labo Service Corporation, INC (Tokyo, Japan). Thirty six mice were divided into six groups (n = 6/group). During the experimental period, the control diet groups (Control groups) were fed D12450J (RESEARCH DIETS INC., Boston, MA, USA) and high-fat diet groups (HFD group) were fed D12492 (RESEARCH DIETS INC.) for 6 weeks. Control and HFD groups were further divided into three subgroups: pure water, 40% (v/v) coffee extract, and 60% (v/v) coffee extract. All groups were allowed free access to food, water, and coffee extract. Body weights and the amounts of food consumed and water and coffee extract drank were measured twice a week for 6 weeks. During the experiments, we monitored food intake and drinking intakes by weighing the food box including control diet or HFD and the bottles containing water or coffee extract twice a week, and calculated the reduction of each box and bottle. We evaluated their weight reductions as the food intake and drinking intake, respectively. All experimental protocols were approved by the Animal Usage Committee of Keio University (Approval number, 15029-(0)). The methods were carried out in accordance with the approved guidelines.

### Measurement of blood glucose levels and free fatty acid, triglyceride, cholesterol and insulin levels in plasma

Mice had been fed the diets and coffee extract for 6 weeks. After the mice were fasted for 12 hr, blood was collected immediately from the inferior vena cava using heparinized syringes under anesthesia by continuous inhalation of 2% isoflurane via a nose cone. Mice were then sacrificed by an overdose of isoflurane. Glucose in whole blood was measured using GLUCOCARD 01 (ARKREY, Minneapolis, MN, USA). Plasma samples were separated by centrifugation (2,000 × g for 15 min) of whole blood with heparin, and were stored at −80°C before analysis. The plasma level of free fatty acids was measured with NEFA (Free Fatty Acid) Reagent (Wako, Osaka, JAPAN). Triglyceride and cholesterol plasma levels were measured with SPOTCHEM II (ARKRAY). The plasma level of insulin was measured with Ultra Sensitive Mouse Insulin ELISA Kit (Morinaga, Yokohama, JAPAN). The methods were carried out in accordance with the approved guidelines.

### Cell culture

Mouse 3T3-L1 preadipocytes were cultured at 37°C with 5% CO_2_ enriched air in DMEM (Nacalai Tesque, Tokyo, Japan) supplemented with 10% fetal bovine serum (FBS) (BioWest, Nuaillé, France), 100 units/mL penicillin (Nacalai Tesque), and 100 μg/mL streptomycin (Nacalai Tesque). In order to investigate the effect of coffee extract on the differentiation into adipocytes, confluent 3T3-L1 cells were treated with culture medium containing 5 mM IBMX, 1 μM dexamethasone, and 10 μg/mL insulin (MDI) in the absence and presence of 5% (v/v) coffee extract. After 2 days, the medium containing MDI was aspirated and replaced by fresh culture medium supplemented with 10 μg/mL insulin with/without 5% (v/v) coffee extract, and the cells were cultured for another 2 days. For further culture of cells, the medium containing insulin was aspirated and replaced by fresh culture medium supplemented with 10 μg/mL insulin with/without 5% (v/v) coffee extract, and the cells were cultured for 2 days as previously described [10, 11, 20].

### Oil-red O staining and quantification

3T3-L1 cells (3×10^4^/well) were seeded on 96-well plates and adipocyte differentiation was induced for 6 days. Cells were washed with phosphate-buffered saline (PBS), fixed with 10% formalin for 30 min, and then washed with 60% isopropanol. After air drying, 0.3% Oil-red O solution (CAYMAN CHEMICAL COMPANY. Ann Arbor, MI, USA) was added to each well at room temperature for 30 min. After the solution was removed, cells were washed with distilled water and air dried. Oil-red O in triglyceride droplets was extracted with 100% isopropanol and determined at OD_510_, as previously described, for quantification [20].

### Reagents and antibodies

Insulin, 3-isobutyl-1-methylxanthine (IBMX), and dexamethasone were purchased from CAYMAN CHEMICAL COMPANY. Dimethyl sulfoxide (DMSO) and cycloheximide (CHX) were purchased from Nacalai Tesque (Tokyo, Japan). MG132 was purchased from Calbiochem (San Diego, CA, USA). An anti-phospho-MEK1/2 antibody (S217/221), anti-MEK1/2 antibody, anti-phospho-ERK1/2 antibody (T202/Y204), anti-ERK1/2 antibody, anti-phospho-Akt antibody (S473), anti-Akt antibody, anti-phospho-Insulin receptor β (IRβ) antibody (Y1146), anti-IRβ antibody, anti-phospho C/EBPβ antibody (T235), anti-Insulin receptor substrate 1 (IRS1) antibody, and anti-IRS2 antibody were purchased from Cell Signaling Technology (Danvers, MA, USA). An anti-C/EBPδ antibody, anti-C/EBPβ antibody, anti-C/EBPα antibody, anti-PPARγ antibody, and anti-β-actin antibody were purchased from Santa Cruz Biotechnology, Inc. (Santa Cruz, CA, USA).

### Immunoprecipitation and immunoblotting

Cell lysates were prepared with Nonidet P-40 lysis buffer (50 mM Tris-HCl, pH 7.4, 10% glycerol, 50 mM NaCl, 0.5% sodium deoxycholate, 0.5% Nonidet P-40, 20 mM NaF, and 0.2 mM Na_3_VO_4_) supplemented with protease inhibitors. Fat tissues were homogenized in Nonidet P-40 lysis buffer supplemented with protease inhibitors. Cell lysates were immunoprecipitated with the anti-IRS1 antibody with the protein G-Sepharose (Zymed Laboratories, South San Francisco, CA, USA) at 4°C for 2 hr. Immunoprecipitates were washed three times with Nonidet P-40 lysis buffer. Denatured samples were resolved on 10% SDS-PAGE and transferred to PVDF membranes. Immunoblotting was performed as previously described [21].

### RNA isolation and Reverse Transcription-Polymerase Chain Reaction (RT-PCR)

RNA was prepared using an RNA purification kit (Qiagen, Hilden, Germany). The reaction of reverse transcription was performed using an oligo (dT)_20_ primer and 2 μg total RNA for first-strand cDNA synthesis, as described previously [21]. Quantitative real-time PCR was performed using an iCycler detection system (Bio-Rad, Berkeley, CA, USA). PCR was performed in a volume of 25 μL with SYBR Green Supermix, SsoFast EvaGreen (Bio-Rad). The PCR primer sequences used were as follows: *C/ebpβ*, 5′-ACAGCGACGAGTACAAGATCC-3′ (upstream) and 5′- GACAGTTGCTCCACCTTCTTCT-3′ (downstream); *C/ebpδ*, 5′-ATCGACTTCAGCGCCTACAT-3′ (upstream) and 5′-GCTTTGTGGTTGCTGTTGAA -3′ (downstream); *Pparγ*, 5’-AACTCTGGGAGATTCTCCTGTTGA-3’ (upstream) and 5’-TGGTAATTTCTTGTGAAGTGCTCATA-3’ (downstream); *C/ebpα*, 5′-AGGTGCTGGAGTTGACCAGT-3′ (upstream) and 5′-CAGCCTAGAGATCCAGCGAC-3′ (downstream); *Cdc45l*, 5’-AAGGGGAATCTGCGAGAAAT-3’ (upstream) and 5’-GGCCAGGAATTTATGCTTGA-3’ (downstream); *Mcm3*, 5’-TGAGCAAGACTGTGGACCTG-3’ (upstream) and 5’-CTTCCTCCTTTTCCGCTTCT-3’ *Gins1*, 5’-CTGGACGAGGGGATCTGATA-3’ (upstream) and 5’-CCCATATTCCCACCTGAGTG-3’ (downstream); *cdc25*, 5’-CCATTCAGATGGAGGAGGAA-3’ (upstream) and 5’-TTTAAGGCTCCCAGGATGTG-3’ (downstream); *Irs1*, 5’-CCAGCCTGGCTATTTAGCTG-3’ (upstream) and 5’-CCCAACTCAACTCCACCACT-3’ (downstream); *Irs2*, 5’-GTAGTTCAGGTCGCCTCTGC-3’ (upstream) and 5’-CAGCTATTGGGACCACCACT-3’ (downstream); *Irβ*, 5’-TCCTGGATTCTGTGGAGGAC-3’ (upstream) and 5’-ATGGTTGGGCAAACTTTCTG-3’ (downstream); *β-actin*, 5′- TGTCCACCTTCCAGCAGATGT -3′ (upstream) and 5′- AGCTCAGTAACAGTCCGCCTAGA-3′ (downstream).

### Cell cycle analysis

After being treated, cells were fixed with 70% (v/v) ethanol at -20°C overnight. Cells were then centrifuged at 5,000 rpm for 2 min and resuspended in PBS containing 10 μg/mL RNase A (Wako, Tokyo, Japan) and 100 μg/ml propidium iodide (Sigma). Cell cycle parameters were determined by a flow cytometric analysis using FACSCalibur [21]. All data were recorded and analyzed using CellQuest software.

### Statistical analysis

Statistical evaluations of the data were expressed as the mean ± S.D. The statistical significance of the differences between the mean values for the treatment groups was analyzed by Student’s *t*-test and one- or two-way analysis of variance (ANOVA) followed by Tukey’s test. Differences were considered to be significant for values of *P*<0.05.

## Results

### Coffee intake effectively prevents HFD-induced obesity

**Fig 1:**
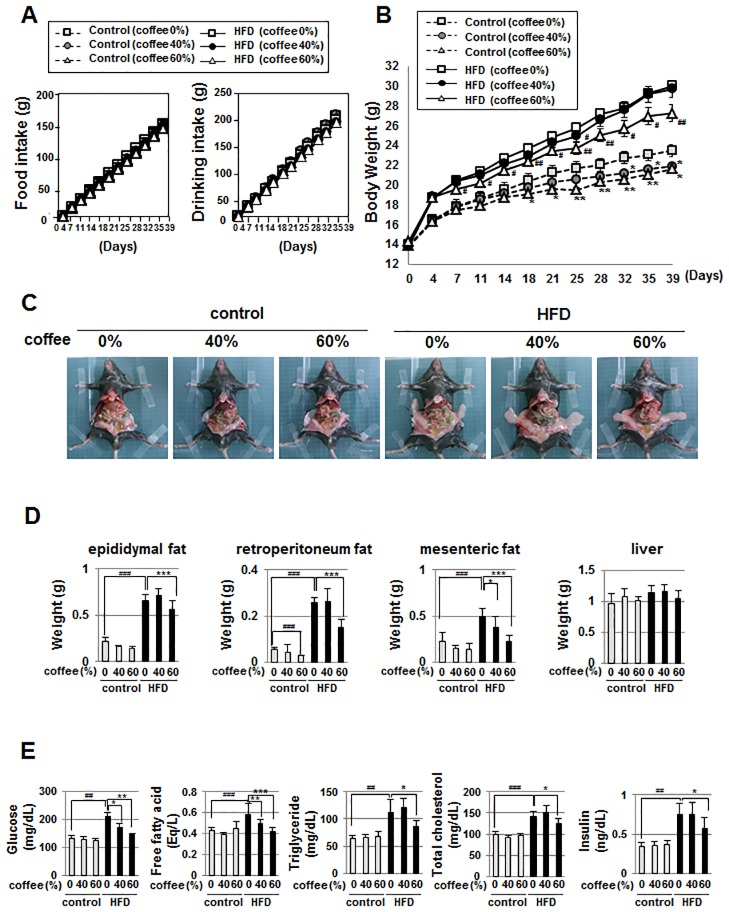
Effects of coffee intake on HFD-induced obesity in C57BL/6 mice. Male C57BL/6 mice were grouped into two groups fed a control diet or high-fat diet (HFD), and were further divided into three subgroups treated with water, 40% (v/v) coffee extract, or 60% (v/v) coffee extract for 6 weeks. (n = 6 mice/group) (A) Cumulative food consumption and drinking intake was graphed. (B) Body weight was monitored twice a week. Data are presented as the mean ± SD. *p < 0.05; **p < 0.01 significantly different from the control diet group given water. #*p* < 0.05; ##*p* < 0.01 significantly different from the HFD diet group given water. (C) In order to show the accumulation of adipose tissue, mice were abdominally opened and photographed. (D) The weights of epididymal fat, retroperitoneum fat, mesenteric fat, and the liver were measured. Data are presented as the mean ± SD. ###*p*<0.001 vs. control (coffee 0%); *p*<0.05, ***p<0.001 vs. HFD (coffee 0%). (E) After six weeks of feeding, mice were fasted for 12 hr and then blood was collected. Blood glucose levels and plasma free fatty acid, triglyceride, and total cholesterol levels were measured and graphed. Data are presented as the mean ± SD. ##*p*<0.05, ###*p*<0.001 vs. control (coffee 0%); *p<0.05, **p<0.01, ***p<0.001, vs. HFD (coffee 0%).

In order to establish the assay system for HFD-induced obesity, male C57BL/6 mice were randomized into two groups, which were fed with either the control diet or HFD. Control diet and HFD groups were divided into three subgroups to further examine the effects of coffee intake on HFD-induced obesity: pure water, 40% (v/v) coffee extract, and 60% (v/v) coffee extract. Intake of 40% (v/v) coffee extract and 60% (v/v) coffee extract were almost same as intake of pure water, and coffee intake did not affect consumption of the control diet or HFD (Fig 1A). As shown in Fig 1B, body weights were markedly higher in mice fed the HFD than in those fed the control diet. Intake of both concentrations (40% (v/v) and 60% (v/v)) of coffee significantly reduced the increase in body mass in the control diet group. The effects of coffee intake on HFD-induced elevations in body weight were then investigated. Intake of 60% (v/v) coffee extract resulted in the reduction of body mass elevations in the HFD-fed groups (Fig 1B). As expected, the accumulation of adipose tissue such as epididymal fat, retroperitoneum fat, and mesenteric fat was greater in mice fed the HFD than in those fed the control diet (Fig 1C and 1D). Coffee intake effectively suppressed the HFD-induced accumulation of adipose tissue, but in a concentration-dependent manner; high concentrations of coffee extract such as 60% (v/v), but not 40% (v/v) were required for this effect. On the other hand, liver weight was not affected by any of these diets (Fig 1D). Blood glucose levels and free fatty acid, triglyceride, total cholesterol and insulin levels in plasma were markedly increased in mice fed the HFD than in those fed the control diet. While the group treated with 40% (v/v) coffee extract showed reductions in blood glucose levels and plasma free fatty acid levels, the intake of 60% (v/v) coffee extract significantly reduced not only blood glucose and plasma free fatty acid levels, but also plasma triglyceride, total cholesterol and insulin levels (Fig 1E). Taken together, these results suggest that coffee intake effectively prevented HFD-induced obesity.

### Coffee extract inhibits MDI-induced adipogenesis in 3T3-L1 preadipocytes

**Fig 2:**
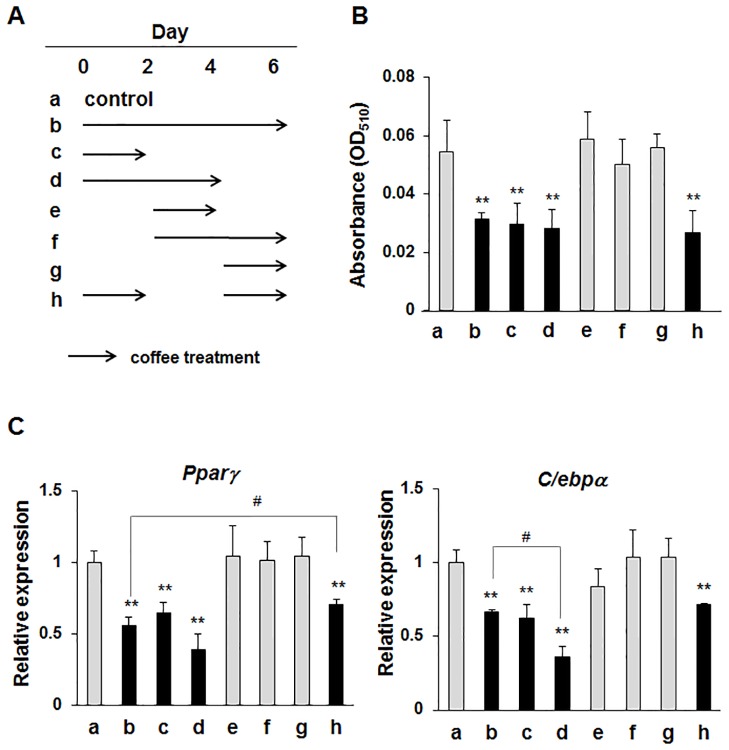
Effects of coffee extract on MDI-induced adipogenesis in 3T3-L1 cells. (A) A time schedule for the treatment with coffee extract during the cellular differentiation of 3T3-L1 cells. 3T3-L1 cells were cultured with medium containing MDI with/without 5% (v/v) coffee extract for 2 days. Then the culture medium was replaced with fresh medium containing insulin with/without 5% (v/v) coffee extract and the cells were cultured for another 2 days. The cells were further cultured with fresh medium containing insulin with/without 5% (v/v) coffee extract for another 2 days. (B) Cells were stained by Oil Red O on day 6 after the MDI treatment. Oil Red O staining was quantified by measuring absorbance at 510 nm (A_510_). Values are the mean ± S.D. of three independent experiments. **p<0.01 vs. control cells (a). (C) Total RNA was prepared on day 6 and the mRNA expression of *Pparγ* and *C/ebpα* was analyzed by quantitative real-time PCR. *β-actin* mRNA was analyzed as an internal control. Values are the mean ± S.D. of three independent experiments. **p<0.01 vs. control cells (a); #*p*<0.05 vs. treated cells (b).

In the previous study, we reported that the coffee extract reduced MDI-induced lipid accumulation in 3T3-L1 preadipocytes [20]. Combining this report with our current observation shown in Fig 1, it is suggested that the anti-obesity activity of coffee intake might be due to the inhibition of adipogenesis by coffee extract. Therefore, we treated 3T3-L1 cells with coffee extract at different stages of cellular differentiation as indicated in Fig 2A to identify the key stage at which coffee extract exerts its anti-adipogenic effects.

Adipogenesis was assessed by Oil Red O staining of lipid droplets and lipid contents, and lipid accumulation was quantified by measuring absorbance at 510 nm. Under the conditions of b, c, d, and h, coffee extract significantly reduced MDI-triggered lipid accumulation, suggesting that it exhibits anti-adipogenic effects within the first 2 days (Fig 2B). Furthermore, the effects of coffee extract on the MDI-induced expression of the master regulators of adipogenesis such as *Pparγ* and *C/ebpα* were determined by RT-PCR. The expression levels of *Pparγ* and *C/ebpα*were also significantly decreased by the treatment with coffee extract within 2 days (Fig 2C).

### Coffee extract inhibits MDI-induced activation of C/EBPβ in 3T3-L1 preadipocytes

**Fig 3:**
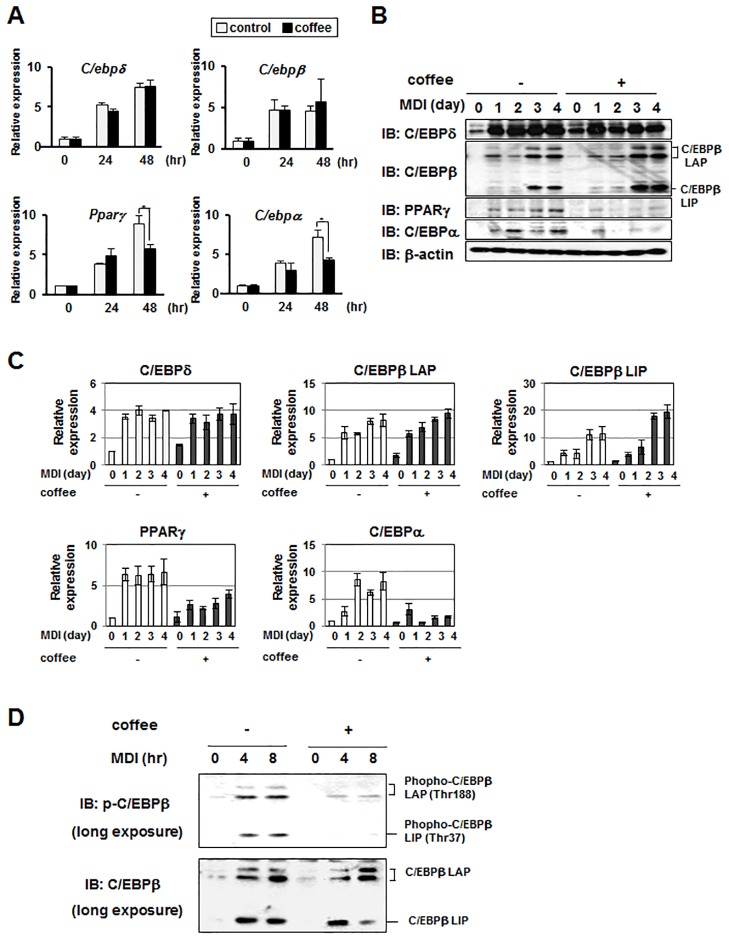
Effects of coffee extract on MDI-induced expression of C/EBP family members and PPARγ in 3T3-L1 cells. (A) 3T3-L1 cells were treated with MDI in the absence and presence of 5% (v/v) coffee extract for 48 hr. Total RNA was prepared and the mRNA expression of *C/ebpδ*, *C/ebpβ*, *Pparγ*, and *C/ebpα* was analyzed by quantitative real-time PCR. *β-actin* mRNA was analyzed as an internal control. Values are the mean ± S.D. of three independent experiments. **p<0.01 vs. control cells. (B) 3T3-L1 cells were treated with MDI in the absence and presence of 5% (v/v) coffee extract for 2 days and then treated with insulin in the absence and presence of 5% (v/v) coffee extract for another 2 days. Whole cell lysates were immunoblotted with an anti-C/EBPδ antibody, anti-C/EBPβ antibody, anti-PPARγ antibody, anti-C/EBPα antibody, or anti-β-actin antibody as a control. LAP and LIP are the translated isoforms of C/EBPβ. (C) The expression levels of C/EBPδ, C/EBPβ LAP, C/EBPβ LIP, PPARγ and C/EBPα were normalized with the expression level of β-actin. The relative expression level of each protein is shown in the graphs. Values are the mean ± S.D. of three independent experiments. **p<0.01, ***p<0.001 vs. control cells. (D) 3T3-L1 cells were treated with MDI in the absence and presence of 5% (v/v) coffee extract for 8 hr. Whole cell lysates were immunoblotted with an anti-phospho-C/EBPβ antibody or anti-C/EBPβ antibody. (E) The phosphorylation levels of C/EBPβ LAP and C/EBPβ LIP were normalized with their protein amounts. Values are the mean ± S.D. of three independent experiments. *p<0.05, **p<0.01 vs. control cells.

Since adipocyte differentiation is regulated by a transcriptional cascade including transcription factors such as C/EBPδ, C/EBPβ, PPARγ and C/EBPα, the effects of coffee extract on the expression of these transcription factors were examined. While coffee extract had no effect on the MDI-induced expression of *C/ebpδ*mRNA or *C/ebpβ* mRNA, it effectively reduced the expression of *Pparγ* mRNA and *C/ebpα* mRNA (Fig 3A). It is well established that C/EBPβincludes several translated isoforms such as LAP (38/35 kDa) and LIP (20 kDa). It has been known that LAP acts as a transcriptionally active isoform of C/EBPβ and LIP functions as an inhibitor of the other C/EBPs by forming non-functional heterodimers, respectively [22, 23]. Consistently, coffee extract specifically inhibited the MDI-induced protein expression of PPARγ and C/EBPα, but not that of C/EBPδ. Without stimulation with MDI, weak expression of C/EBPβ LAP was detected in the undifferentiated 3T3-L1 cells, however the expression of C/EBPβ LIP was expressed under the detectable level, nevertheless the presence or absence of coffee extracts. Once cells were sequentially treated with MDI and insulin for the adipogenesis, the expressions of both LAP and LIP were drastically increased after three days of the sequential treatment with MDI and insulin (Fig 3B and 3C). The MDI-induced expression of C/EBPβ LIP was significantly enhanced by coffee extract (Fig 3B and 3C). Since *Pparγ* and *C/ebpα* are target genes of C/EBPβ [4–7], coffee extract may inhibit the transcriptional activity of C/EBPβ. The phosphorylation of C/EBPβ at Thr-235 is essential for its transcriptional activity [24]; therefore, we investigated the effects of coffee extract on the phosphorylation of C/EBPβ at Thr-235 by performing an immunoblot analysis. Since we attempted to analyze the initial effect of MDI on the expression and the phosphorylation of C/EBPβ, we investigated it by utilizing longer exposure film for the immunoblot (Fig 3D). As shown in Fig 3D and 3E, MDI induced not only the expression of C/EBPβ, but also its phosphorylation. Coffee extract significantly reduced the MDI-induced phosphorylation of C/EBPβ. These results indicate that coffee extract suppresses adipogenesis by inhibiting the activation of C/EBPβ.

### Coffee extract inhibits MDI-induced cell cycle progression during the differentiation of 3T3-L1 preadipocytes

**Fig 4:**
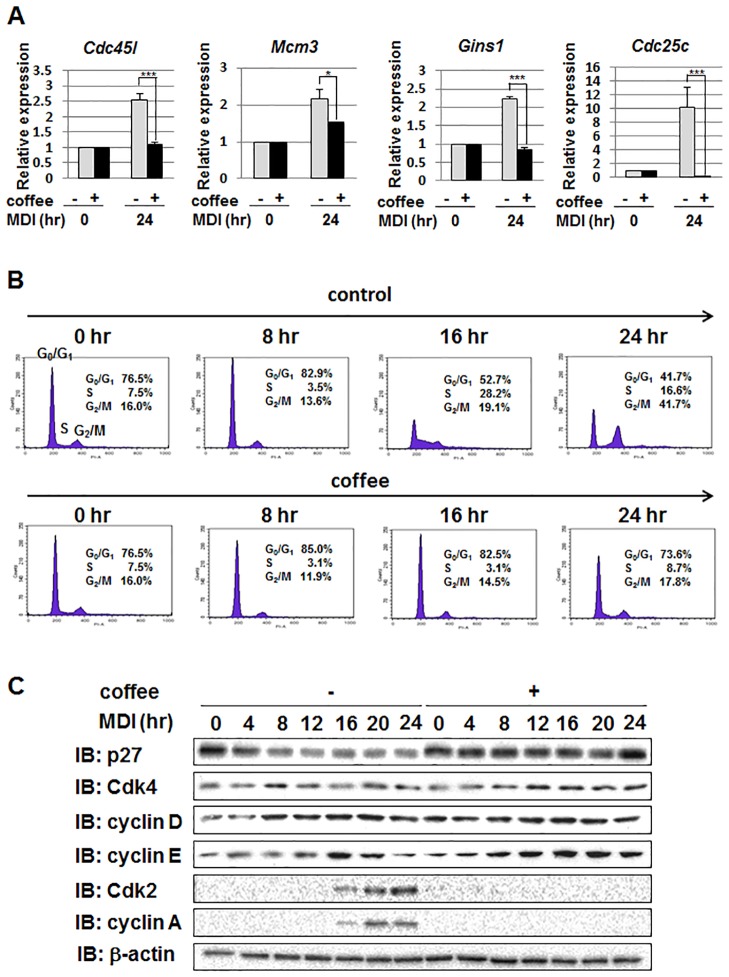
Effects of coffee extract on MDI-induced cell cycle progression in 3T3-L1 cells. 3T3-L1 cells were treated with MDI in the absence and presence of 5% (v/v) coffee extract for the indicated periods. (A) After the stimulation with MDI for 24 hr, total RNA was prepared and the mRNA expression of *Cdc45l*, *Mcm3*, *Gins1*, and *Cdc25c* was analyzed by quantitative real-time PCR. *β-actin* mRNA was analyzed as an internal control. Values are the mean ± S.D. of three independent experiments. *p<0.05, ***p<0.001 vs. control cells. (B) Cells were fixed and treated with propidium iodide (PI). The population of cells in each stage of the cell cycle was quantified using a flow cytometer. (C) Whole cell lysates were prepared and immunoblotted with an anti-p27 antibody, anti-Cdk4 antibody, anti-cyclin D antibody, anti-cyclin E antibody, anti-Cdk2 antibody, anti-cyclin A antibody, or anti-β-actin antibody.

MCE is a necessary step for terminal differentiation into adipocytes [16]. C/EBPβ plays a crucial role in MCE by inducing the expression of its target genes such as *Cdc45l*, *Mcm3*, *Gins1*, and *Cdc25* [25–27]. We investigated the effects of coffee extract on the expression of these genes using RT-PCR. As shown in Fig 4A, MDI significantly induced the expression of *Cdc45l*, *Mcm3*, *Gins1*, and *Cdc25* mRNAs. On the other hand, coffee extract effectively inhibited the expression of these mRNAs induced by the MDI stimulation (Fig 4A). The effects of coffee extract on MDI-induced cell cycle progression were analyzed by performing a flow cytometric analysis. MDI promoted the cell cycle progression of growth-arrested 3T3-L1 cells, and their entry into the S and G2/M phases 16 and 24 hr after the stimulation with MDI, respectively. On the other hand, coffee extract completely inhibited MDI-induced cell cycle progression (Fig 4B). We also investigated the effects of coffee extract on the expression levels of cell cycle regulators. The cyclin-dependent kinase inhibitor, p27^kip1^ has been shown to inhibit cell cycle progression from the G0 to G1 phase [28]. As shown in Fig 4C, the stimulation with MDI markedly reduced the protein expression of p27^kip1^; however, coffee extract significantly inhibited the reduction of p27^kip1^, supporting the results in Fig 4B. Coffee extract had a negligible effect on the expression of Cdk4 and cyclin D in cells treated with MDI. Whereas the expression of cyclin E was induced 16 hr after the treatment with MDI and decreased thereafter in control cells, the induced expression of cyclin E did not decrease until 24 hr in cells treated with coffee extract. In addition, the MDI-induced expression of Cdk2 and cyclin A was completely inhibited by coffee extract. Collectively, these results suggest that coffee extract inhibits cell cycle entry into the S phase of 3T3-L1 cells and also potentially the initiation of MCE, and appears to be one of the mechanisms by which coffee extract suppresses adipogenesis.

### Coffee extract inhibits MDI-induced activation of the MEK-ERK pathway and Akt in 3T3-L1 preadipocytes

**Fig 5:**
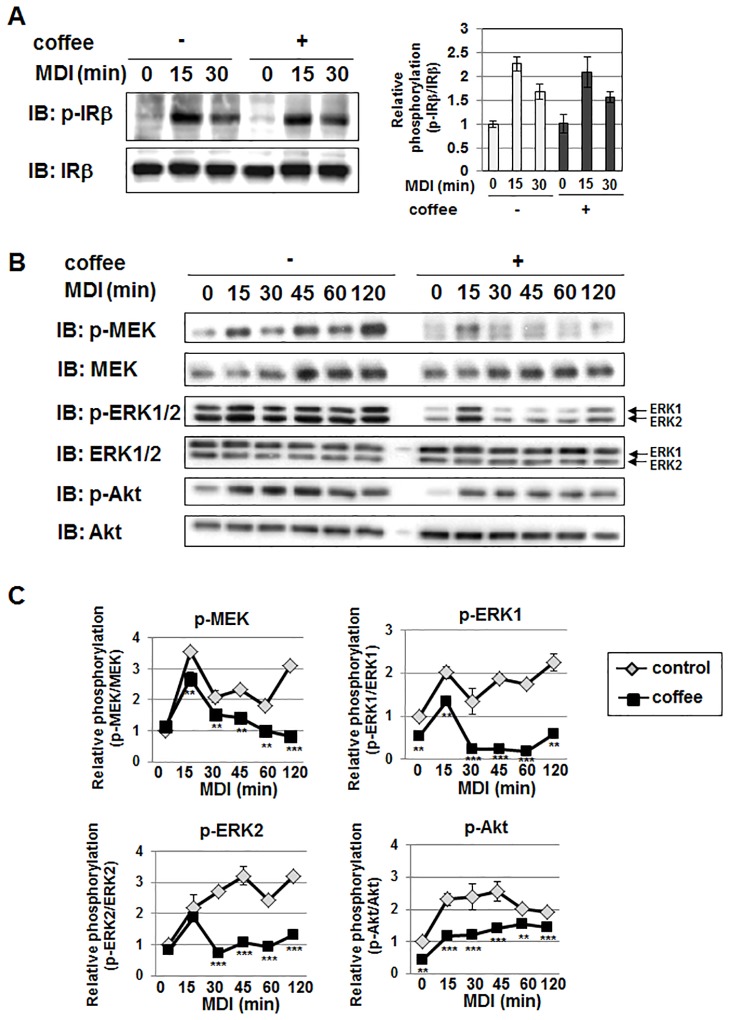
Effects of coffee extract on MDI-induced phosphorylation of IRβ, MEK, ERK, and Akt in 3T3-L1 cells. 3T3-L1 cells were treated with 5% (v/v) coffee extract for 1 hr, and then stimulated with MDI for the indicated periods. (A) Whole cell lysates were immunoblotted with an anti-phospho-IRβ antibody or anti-IRβ antibody. The relative phosphorylation of IRβ is shown in the graph. Values are the mean ± S.D. of three independent experiments. *p<0.01, ***p<0.001 vs. untreated cells. (B) Whole cell lysates were immunoblotted with an anti-phospho-MEK antibody, anti-MEK antibody, anti-phospho-ERK1/2 antibody, anti-ERK antibody, anti-phospho-Akt antibody, or anti-Akt antibody. (C) The phosphorylation levels of MEK, ERK1, ERK2, and Akt were normalized with their protein amounts. The relative phosphorylation of MEK, ERK1, ERK2, and Akt is shown in the graphs. Values are the mean ± S.D. of three independent experiments.

The insulin signaling pathway contributes to the initiation of MCE during adipogenesis as a critical initiator for cell cycle progression [29–31]. Therefore, we determined whether coffee extract affects the insulin signaling pathway. The stimulation with insulin caused the auto-phosphorylation of the β subunit of the insulin receptor (IRβ) at its tyrosine residues. Similar to the stimulation with insulin alone, the combined stimulation with MDI also caused the phosphorylation of IRβ. However, coffee extract failed to affect the phosphorylation level of IRβ (Fig 5A). On the other hand, it significantly inhibited the MDI-induced activation of MEK, ERK1/2, and Akt (Fig 5B and 5C). These results strongly suggest that coffee extract affects insulin signaling, and appears to be due to the upstream signaling of MEK, ERK1/2, and Akt, but not IRβ.

### Coffee extract reduces the protein expression level of IRS1 in 3T3-L1 preadipocytes

**Fig 6:**
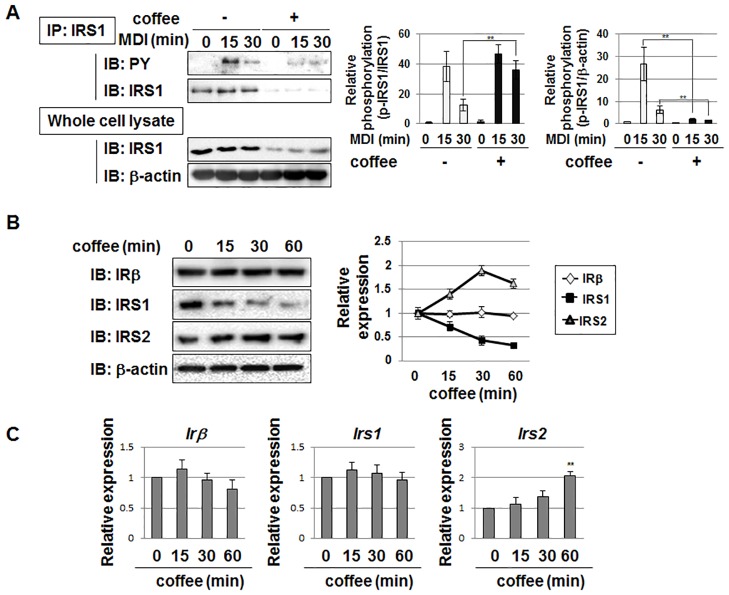
Effects of coffee extract on expression of IRβ, IRS1, and IRS2 in 3T3-L1 cells. (A) 3T3-L1 cells were treated with 5% (v/v) coffee extract for 1 hr, and then stimulated with MDI for the indicated periods. Cell lysates were immunoprecipitated (IP) with an anti-IRS1 antibody, followed by immunoblotting (IB) with an anti-phospho-tyrosine (PY) antibody or anti-IRS1 antibody. Whole cell lysates were immunoblotted with an anti-β-actin antibody. The phosphorylation level of IRS1 was normalized with the expression level of IRS1 or the expression level of β-actin. The relative phosphorylation of IRS1 is shown in the graphs. Values are the mean ± S.D. of three independent experiments. **p<0.01 vs. control cells. (B, C) 3T3-L1 cells were treated with 5% (v/v) coffee extract for the indicated periods. (B) Cell lysates were immunoblotted with an anti-IRβ antibody, anti-IRS1 antibody, anti-IRS2 antibody, or anti-β-actin antibody. The expression level of IRβ, IRS1, and IRS2 was normalized with the protein amount of β-actin. The relative expression level of IRβ, IRS1, and IRS2 is shown in the graphs. Values are the mean ± S.D. of three independent experiments. (C) Total RNA was prepared and the mRNA expression of *Irβ*, *Irs1*, and *Irs2* was analyzed using quantitative real-time PCR. *β-actin* mRNA was analyzed as an internal control. Values are the mean ± S.D. of three independent experiments. **p<0.01 vs. untreated cells.

In order to elucidate the mechanisms by which coffee extract inhibits the MDI-induced activation of MEK-ERK and Akt, we investigated whether coffee extract affects the phosphorylation of IRS1 induced by IRβ. IRS1 lies downstream of the insulin receptor, is phosphorylated by activated insulin receptors, and functions as an adaptor protein for the activation of the MEK-ERK pathway and Akt signaling [32, 33]. IRS1 was immunoprecipitated and its tyrosine phosphorylation level was determined by immunoblotting using the anti-phospho-tyrosine antibody. The stimulation with MDI caused the tyrosine phosphorylation of IRS1; however, coffee extract markedly reduced the phosphorylation of IRS1. Significantly, the treatment with coffee extract also reduced the protein amount of IRS1. Furthermore, we calculated the ratio between the phosphorylated IRS1 and the protein amount of IRS1 (pIRS1/IRS1), and this ratio was slightly increased by the coffee extract. On the other hand, when we pay our attention to the ratio between phosphorylated IRS1 and β-actin in whole cell lysate, coffee extract drastically decreased the relative level of IRS1 phosphorylation. These observation suggested that the reduction of IRS1 phosphorylation by coffee extract seems to be due to the down-regulation of IRS1 protein (Fig 6A). The effects of coffee extract on the protein expression of IRβ and the insulin receptor substrate proteins, IRS1 and IRS2 were then examined. Coffee extract did not affect the expression level of IRβ, and slightly increased that of IRS2. However, the expression level of the IRS1 protein was markedly reduced by the treatment with coffee extract (Fig 6B). We analyzed the effects of coffee extract on the mRNA expression of these signaling molecules. Coffee extract did not alter the mRNA levels of *Irβ* or *Irs1*. On the other hand, *Irs2* mRNA levels were slightly increased by the treatment with coffee extract (Fig 6C). These results strongly suggest that coffee extract decreases the expression of IRS1 in a manner that is mediated by post-transcriptional regulation, such as protein synthesis or degradation.

### Coffee extract reduces the stability of IRS1 in 3T3-L1 preadipocytes

**Fig 7:**
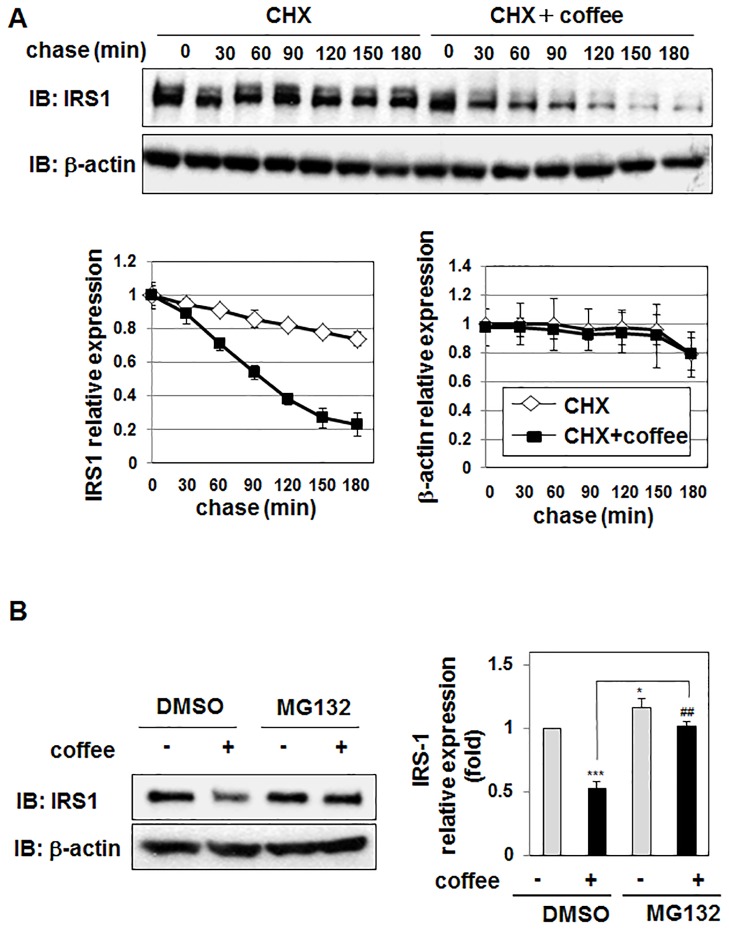
Effects of coffee extract on the stability of IRS1 in 3T3-L1 cells. (A) 3T3-L1 cells were treated with cycloheximide (CHX) (100 μg/mL) in the presence and absence of coffee extract (5% (v/v)) for the indicated periods. Cell lysates were immunoblotted with an anti-IRS1 antibody or anti-β-actin antibody. The expression level of IRS1 and β-actin was quantified and shown in the graphs. Values are the mean ± S.D. of three independent experiments. (B) 3T3-L1 cells were pretreated with DMSO (0.1%) or MG132 (20 μM) for 1 hr prior to the treatment with coffee extract (5% (v/v)) for 1 hr. Cell lysates were immunoblotted with an anti-IRS1 antibody or anti-β-actin antibody. The expression level of IRS1 was normalized to the protein amount of β-actin. The relative expression level of IRS1 is shown in the graphs. Values are the mean ± S.D. of three independent experiments. *p<0.05; ***p<0.001 significantly different from the control group treated with DMSO. ##*p* < 0.01 significantly different from the group treated with DMSO and coffee.

In order to gain further insights into the mechanisms by which coffee extract induces the down-regulation of IRS1, we investigated the effects of coffee extract on the degradation of IRS1 in the presence of an inhibitor of protein synthesis, cycloheximide (CHX). The time-dependent reduction of the IRS1 protein was markedly faster in cells treated with the combination of CHX and coffee extract than in those treated with CHX alone, suggesting that coffee extract reduces the stability of the IRS1 protein. On the other hand, coffee extract did not affect the stability of β-actin (Fig 7A). In order to determine whether coffee extract promotes the degradation of IRS1 via the proteasomal pathway, we examined the effects of the proteasomal inhibitor, MG132, on the expression of the IRS1 protein. The treatment with MG132 effectively canceled coffee extract-induced decreases in the expression of IRS1, suggesting that coffee extract induces the degradation of IRS1 by a proteasomal pathway (Fig 7B).

### Coffee intake reduces the protein expression level of IRS1 in adipose tissues

**Fig 8:**
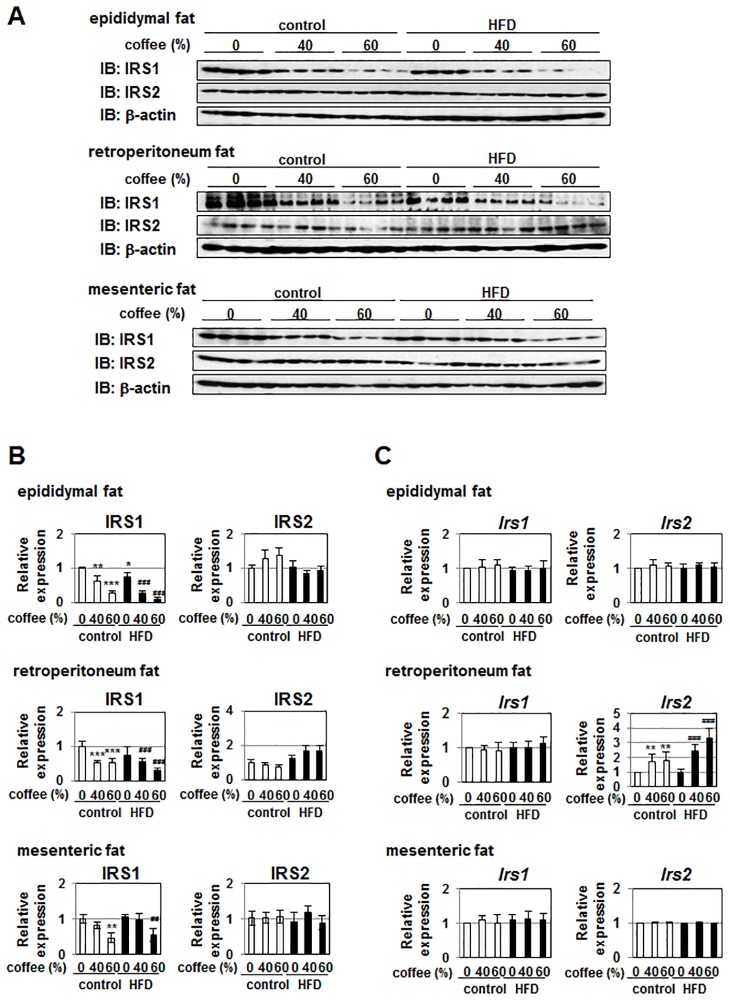
Effects of coffee intake on expression of IRS1 in C57BL/6 mice fed with control diet or HFD. Male C57BL/6 mice were grouped into two groups fed a control diet or HFD, and were further divided into three subgroups treated with water, 40% (v/v) coffee extract, or 60% (v/v) coffee extract for 6 weeks. (n = 4 mice/group) (A) Fat tissues such as epididymal fat, retroperitoneum fat and mesenteric fat were homogenized and immunoblotted with anti-IRS1 antibody, anti-IRS2 antibody or anti-β-actin antibody. (B) The protein expression level of IRS1, and IRS2 was normalized with the protein amount of β-actin. The relative expression level of IRS1, and IRS2 is shown in the graphs. Data are presented as the mean ± SD. *p<0.05; **p<0.01; ***p<0.001 significantly different from the control diet group given water. ##*p* < 0.01: ###*p*<0.001 significantly different from the HFD group given water. (C) Total RNA was prepared from epididymal fat, retroperitoneum fat and mesenteric fat and the mRNA expression of *Irs1* and *Irs2* was analyzed using quantitative real-time PCR. *β-actin* mRNA was analyzed as an internal control. Data are presented as the mean ± SD. **p<0.01 significantly different from the control diet group given water. ###*p*<0.001 significantly different from the HFD group given water.

Finally, we examined the effect of coffee intake on protein expression level of IRS1 in adipose tissues of mice fed the control diet and HFD. Because of difficulty to extract the protein from adipose tissues, we only could extract the both protein and RNA samples from four individual mice. Then, we performed the immunoblotting using successfully extracted four individual samples from each adipose tissue. In epididymal fat, retroperitoneum fat and mesenteric fat, protein expression level of IRS1 tended to be lower in mice fed the HFD than those fed the control diet. Strikingly, coffee intake effectively reduced the protein expression level of IRS1 in a concentration-dependent manner both in mice fed the control diet and the HFD. On the other hand, the protein expression level of IRS2 in epididymal fat, retroperitoneum fat and mesenteric fat was little affected by HFD and coffee intake (Fig 8A and 8B). Furthermore, the expression of *Irs1* mRNA level hardly changed by intake of HFD and coffee. Expression level of *Irs2* mRNA was not changed by HFD. Although coffee intake had no effect on protein expression of IRS2, the expression level of *Irs2* mRNA in retroperitoneum fat was significantly increased by coffee intake in a dose dependent manner. On the other hand, the expression level of *Irs2* mRNA in epididymal fat and mesenteric fat were not affected by coffee intake (Fig 8C). Taken together, it is suggested that coffee extract reduced the protein expression of IRS1 not only in 3T3-L1 cells but also in adipose tissues *in vivo*.

## Discussion

**Fig 9:**
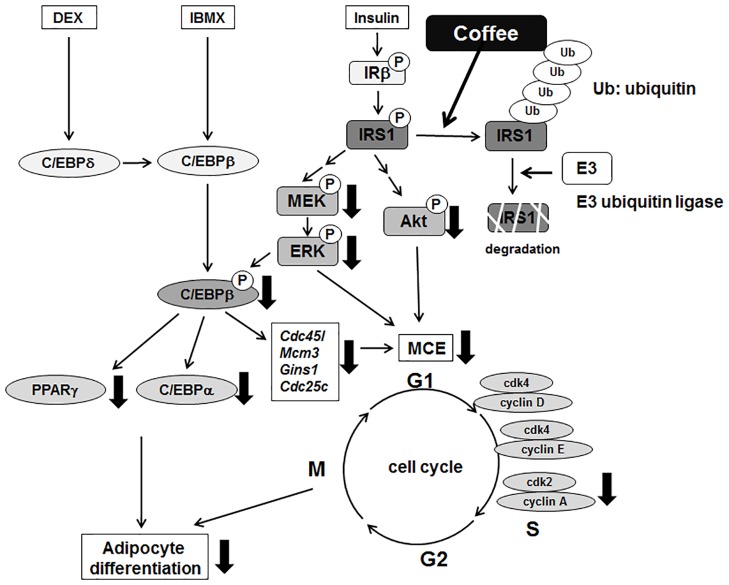
Inhibitory mechanism of adipogenesis by coffee extract. Coffee extract reduces the expression of the IRS1 protein by proteasomal degradation, thereby downregulating the activation of the MEK-ERK and Akt pathways. Thus, in cells treated with coffee extract, the phosphorylation of C/EBPβ mediated by ERK is inhibited and C/EBPβ is inactivated, which inhibits the expression of its target genes such as *Pparγ*, *C/ebpα*, *Cdc45l*, *Mcm3*, *Gins1*, and *Cdc25*, which are required for adipogenesis. E3 indicates E3 ubiquin ligase. Ub indicates ubiquitin.

In the present study, we analyzed the molecular mechanism by which coffee extract inhibited adipogenesis in 3T3-L1 cells to understand the anti-obesity activity of coffee extract. Previously, we reported that coffee extract inhibited the expression of *Pparγ* which led to reduced gene expression of adipocyte markers such as *Fabp4*, *Adiponectin*, *Glut4*, and *Lpl* [20]. In the current study, we found that coffee extract reduced the activity of C/EBPβ, resulting in the inhibition of MCE, which is critical for adipogenesis and the inactivation of PPARγ (Figs 3 and 4). Although coffee extract did not influence the expression of C/EBPβ, the MDI-induced phosphorylation of C/EBPβ, which is mediated by ERK, was decreased in cells treated with coffee extract (Figs 3 and 9. We additionally found that coffee extract reduced the protein expression level of IRS1 through proteasomal degradation, thereby inhibiting the MDI-induced activation of ERK (Fig 7). Through these studies, we concluded that IRS1 is an important target molecule for coffee extract to exhibit anti-obesity effect.

IRS1 and IRS2 are both important adapter molecules in the insulin signaling pathway; however, these two molecules have different physiological functions. The disruption of IRS1 in mice retards increases in body mass and reduces insulin-stimulated glucose uptake; however, diabetes does not develop because insulin secretion increases in order to compensate for mild resistance to insulin [34, 35]. On the other hand, the disruption of IRS2 impaired peripheral insulin signaling and pancreatic β-cell function, and these incomplete signaling pathways finally caused diabetes [36]. In the present study, we showed that the treatment with coffee extract caused the degradation of IRS1, but not IRS2 (Fig 6). Suppressor of cytokine signaling 1/3 (SOCS1/3)-containing E3-ligases were previously shown to target IRS1 and IRS2 for ubiquitin-proteasomal protein degradation [37]. SOCS proteins bind to their target proteins through their SH2 domain, suggesting that the tyrosine phosphorylation of target proteins is required for the interaction between SOCS proteins and their targets [38, 39]. Since the tyrosine phosphorylation of IRS1 was induced by MDI in cells treated with coffee extract (Fig 6A), it was expected that the interaction between IRS1 and SOCS1/3 were caused. However, difficulties are associated with determining the mechanisms by which SOCS1/3 specifically selects IRS1, but not IRS2 as a target protein in coffee-treated cells. On the other hand, Cullin-RING E3 ubiquitin ligase 7 (CUL7) was identified as an IRS1-specific ubiquitin ligase [40]. It has been reported that the phosphorylation of IRS1 induced by S6 kinase (S6K) triggers an interaction between IRS1 and CUL7 for ubiquitin-proteasomal degradation [41]. Although we do not have suitable observation suggesting the involvement of S6K in the coffee extract-caused anti-obesity effect, it will be important to examine the influence of coffee extract on activity of S6K and CUL7, leading to the destabilization of IRS1.

The intake of coffee significantly inhibited HFD-induced increases not only in body weight and the accumulation of adipose tissue, but also in blood glucose levels and plasma free fatty acid, triglyceride, and total cholesterol levels (Fig 1). Coffee intake at a concentration of 60% (v/v) by mice in the present study is equivalent to approximately 6–7 cups of coffee per day by humans. Choi et al. reported that the weights of liver and AST levels in the HFD-fed mice were higher than in the normal diet-fed mice. In addition, they showed that the weights of liver and AST levels in HFD groups were significantly reduced by oral administration of green coffee bean extract [9]. However, in the current study, we could not observe any differences of the weight of liver and AST levels between in the control mice and the mice fed HFD. We could not provide the suitable explanation why we observed the different result from theirs, but it was more likely to be caused by the differences in fat content and fat raw materials of HFD utilized.

Coffee extract contains a large number of chemical elements such as caffeine, chlorogenic acid, and caffeic acid, which are known to be abundant in coffee extract [42]. A previous study reported a negative correlation between coffee consumption and the rate of pathogenesis of type 2 diabetes in groups drinking caffeinated coffee and decaffeinated coffee [43], indicating that other phytochemicals, but not caffeine, are critical for this beneficial bioactivity of coffee beans. Furthermore, chlorogenic acid and caffeic acid were previously reported to prevent the HFD-induced obesity [44, 45]. These findings support the hypothesis that chlorogenic acid and caffeic acid are the active compounds preventing diet-induced obesity; however; we observed that caffeine, chlorogenic acid and caffeic acid failed to inhibit MDI-induced PPARγ expression in 3T3-L1 cells, suggesting that they are not the ingredients of coffee inhibiting adipogenesis [20]. The coffee ingredients that inhibit adipogenesis need to be identified in future studies in order to more clearly elucidate the mechanisms by which coffee intake is beneficial for maintaining metabolic homeostasis.

## Conclusion

In this study, we clarified the mechanism by which coffee extract inhibited the differentiation inducers (MDI)-induced adipogenesis in 3T3-L1 preadipocytes. Coffee extract induced the degradation of IRS1 which is an adaptor molecule in insulin signaling through proteasomal pathway. As a result, coffee extract inhibited the MDI-induced activation of C/EBPβ by preventing its phosphorylation by ERK, leading to suppression of the adipogenesis-related events such as mitotic clonal expansion (MCE) and the expression of *Pparγ* and *C/ebpα*. Furthermore, coffee intake significantly prevents the HFD-induced obesity in mice and reduced the protein expression level of IRS1 in adipose tissues. Considering these results, it was revealed that IRS1 is a novel target for coffee extract in adipogenesis.

## Supporting information

S1 Fig Supplementary information. Photographs of the full-length blots utilized in Figures.
(TIF)

## Abbreviations

C/EBP: CCAAT/enhancer-binding protein
Cdc25c: cell division cycle 25 homolog c
Cdc45l: cell division cycle 45 homolog
CHX: cycloheximide
CUL7: cullin-RING E3 ubiquitin ligase 7
ERK: extracellular signal-regulated kinase
GINS: go-ichi-ni-san
Gins1: GINS complex subunit 1
HFD: high-fat diet
IBMX: 3-isobutyl-1-methylxanthine
IRβ: Insulin receptor β
IRS: Insulin receptor substrate
MCE: mitotic clonal expansion
MCM2–7: mini-chromosome maintenance proteins 2–7
Mcm3: mini-chromosome maintenance complex component 3
MDI: IBMX: dexamethasone and insulin
mTORC1: mechanistic target of rapamycin complex 1
PPARγ: peroxisome proliferator-activated receptor γ
S6K: kinase S6 kinase
WAT: white adipose tissue
