# Supplementary figures and images for: Coffee extract inhibits adipogenesis in 3T3-L1 preadipocyes by interrupting insulin signaling through the downregulation of IRS1
Source: PLoS One. 2017 Mar 10;12(3):e0173264. doi: 10.1371/journal.pone.0173264 (PMC5345824; doi:10.1371/journal.pone.0173264)

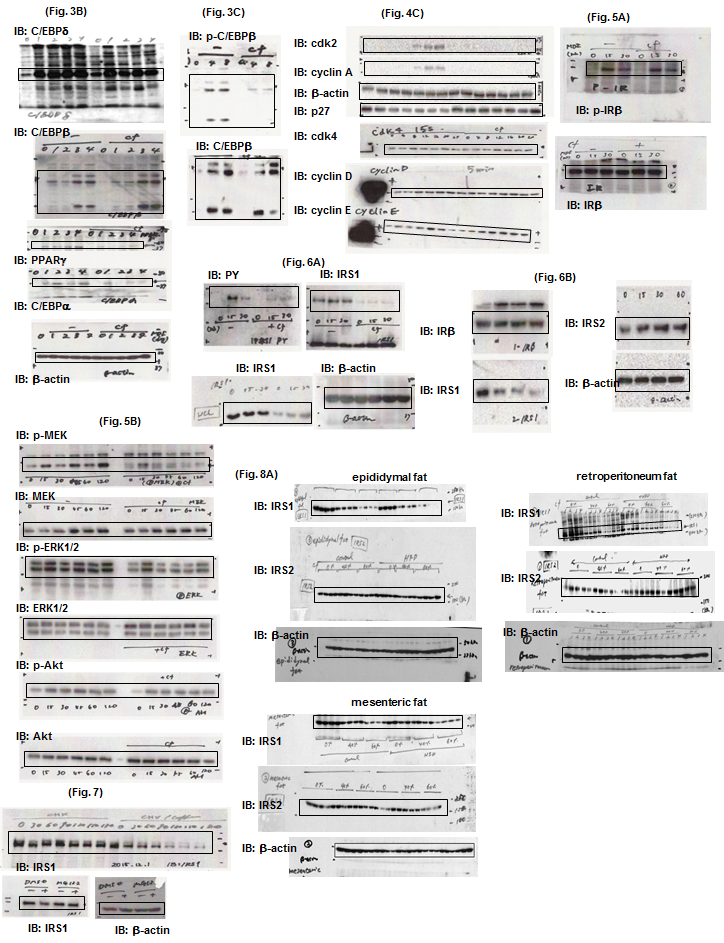

Supplement: S1 Fig — Photographs of the full-length blots utilized in Figures. (TIF) [file pone.0173264.s001.tif]
